# Acrylamide toxicity in aquatic animals and its mitigation approaches: an updated overview

**DOI:** 10.1007/s11356-023-30437-4

**Published:** 2023-10-23

**Authors:** Mohammed A. E. Naiel, Samar S. Negm, Shakira Ghazanfar, Arshad Farid, Mustafa Shukry

**Affiliations:** 1 Department of Animal Production, Faculty of Agriculture, Zagazig University, Zagazig, 44519 Egypt; 2 Fish Biology and Ecology Department, Central Laboratory for Aquaculture Research (CLAR), Abbassa 44661, Agriculture Research Center, Giza, Egypt; 3 National Institute for Genomics Advanced and Biotechnology (NIGAB), National Agricultural Research Centre, Park Road, Islamabad, 45500 Pakistan; 4 Gomal Center of Biochemistry and Biotechnology, Gomal University, D. I. Khan, 29050 Pakistan; 5 Physiology Department, Faculty of Veterinary Medicine, Kafrelsheikh University, Kafr El-Sheikh, 33516 Egypt

**Keywords:** Acrylamide, Environmental contamination, Phytochemical, Fish welfare toxicity, Biodegradation

## Abstract

Acrylamide (ACR) is widely applied in various industrial activities, as well as in the water purification process. Furthermore, ACR is synthesized naturally in some starchy grains exposed to high temperatures for an extended time during the cooking process. Because of its widespread industrial usage, ACR might be released into water stream sources. Also, ACR poses a high risk of contaminated surface and ground-water resources due to its high solubility and mobility in water. Furthermore, animal studies have indicated that ACR exposure may cause cancer (in many organs such as lung, prostate, uterus, and pancreas), genetic damage (in both somatic and germ cells), and severe effects on reproduction and development. Recently, numerous studies have shown that ACR has a mild acute cytotoxic impact on aquatic species, particularly during early life stages. Besides, wide-spectrum usage of ACR in many industrial activities presented higher environmental risks as well as major hazards to consumer health. This literature was designed to include all potential and accessible reports on ACR toxicity related with aquatic species. The Preferred Reporting Items for Systematic Reviews were applied to evaluate the risk effects of ACR on aquatic organisms, the ACR sub-lethal concentration in the ecosystem, and the possible protective benefits of various feed additives against ACR toxicity in fish. The major findings are summarized in Tables 2 and 3. The primary aim of this literature was to specify the hazards of ACR toxicity related with fish welfare and possible suggested strategies to reduce its risks.

## Introduction

Acrylamide (ACR), a monomer with a vinyl group, has neurotoxic effects on the nervous system in both humans and animals such as overall weakness, numbness, tingling in the limbs, or ataxia (Kopańska et al. 2022; Lakshmi et al. 2012). ACR and its components are mostly used as flocculants, and the main areas of application include extraction of inorganic minerals, purifying waste water, and food-processing manufacturing industries (Aras et al. 2017). ACR is effective in purification irrigation and drinking water (Becalski et al. 2003). The ACR applied in the purification treatments and flocculation process can pollute the ambient ecosystem by releasing its residual (Ciesarova et al. 2006). ACR does not have the ability to be adsorbed with the soil and can easily degrade into ground-water via seepage, which might lead to a high risk to public health and welfare (Croll et al. 1974). Therefore, ACR contamination in water is a threat to aquatic wildlife (Tanekhy and Mehana 2022).

The main problem with ACR pollution is the lack of a filtration procedure before its release into water streams or consumption as drinking water (Exon 2006). Besides, many regions across the world, especially in developing countries, have not performed frequent detectable analyses for ACR permissible levels in drinking water, which may threaten public health (Tepe 2015). Furthermore, there is evidence of ACR polluting surface and drinking water supplies worldwide (Tepe and Çebi 2019). Also, the release of ACR into water streams from various uses such as agriculture, herbicides, and cosmetics has been identified as the main issue for ecosystem pollution and threatens fish sustainability (Naiel et al. 2020d; Wang and Lee 2001).

According to toxicological research, ACR is quickly absorbed throughout the body and is even passed on to offspring after accumulating throughout the organism (El-Shehawi et al. 2022; Yue et al. 2021). Several recent studies have shown that ACR causes acute neurotoxicity in the developmental stage of zebrafish when exposed to nearly 1 mM ACR for 3 days (Faria et al. 2018, 2019a; Park et al. 2021). Moreover, ACR could be genotoxic to *Carassius auratus* at levels 50–150 mg/L, shows hemato-toxicity (such as induced anemia, red blood cell count reduction, low hemoglobin level, and decreased packed cell volume) in *Clarias gariepinus* at concentrations from 26.6 up to 106.4 mg/L (Ibrahim and Ibrahem 2020), and induces cardiac developmental toxicity at levels from 71 to 203 mg/L, cardiovascular toxicity at concentrations from 35.5 to 203 mg/L, retinal toxicity (71–142 mg/L), splenic toxicity (12.8 mg/kg), and oxidative stress (142 mg/L) in *Danio rerio* (Moura et al. 2019; Singh et al. 2019; Spencer et al. 2018). Besides, many studies have shown a lethal concentration of ACR in several fish species (Gopika et al. 2018b; Krautter et al. 1986; Larguinho et al. 2014b; Moura et al. 2019; Shanker and Seth 1986; Spencer et al. 2018). Recently, Ligina et al. (2022) exhibited that ACR at sublethal levels (13.4 µg/L) induced alteration in hematological indices and suppressed redox status in gill tissue of the *A. testudineus* fish. The main objective of the current literature was to shed light on the structure and resources of ACR, and its stability in the ecosystem and release into stream water. Furthermore, the main aspects of this literature are identifying the adverse effects of ACR-contaminated water on fish health. Likewise, it recommends novel appropriate and practical techniques to lower and mitigate ACR threats in the ecosystem and fish sustainability via enhanced fish immune system and antioxidant status and degrades ACR compounds through employing microbial degradation methods.

## Methodology

This literature investigated all possible and available studies that were found to be related to ACR toxicity in fish and crustacean. Besides, available *in vivo* or *in vitro* reports have shown the anticipated functional relevance of some feed additives on alleviating ACR toxicity in fish. A comprehensive literature search was directed using the Scopus (2003–2021), Google Scholar, and Web of Science (1900–2021) databases. The search was done using the following search criteria for terms in the title, keywords, or abstract:

(acrylamide* OR toxic* OR hazard* OR phytochemical OR salt* OR pigment* OR herb OR *acid* OR additive* OR diet) AND (fish* OR shellfish* OR crustacean*) AND (immun* OR antioxidant* OR growth* OR oxidative AND* OR production OR meat OR bacterial OR mortality* OR disease*) AND NOT (hot AND water OR methano* OR acetone OR trace* OR metal* OR *icide* OR nano* OR toxic* OR lethal OR fungi OR mammal* OR human OR patient OR cancer OR product* OR tumor OR vaccine*) AND (acrylamide).

**Fig. 1:**
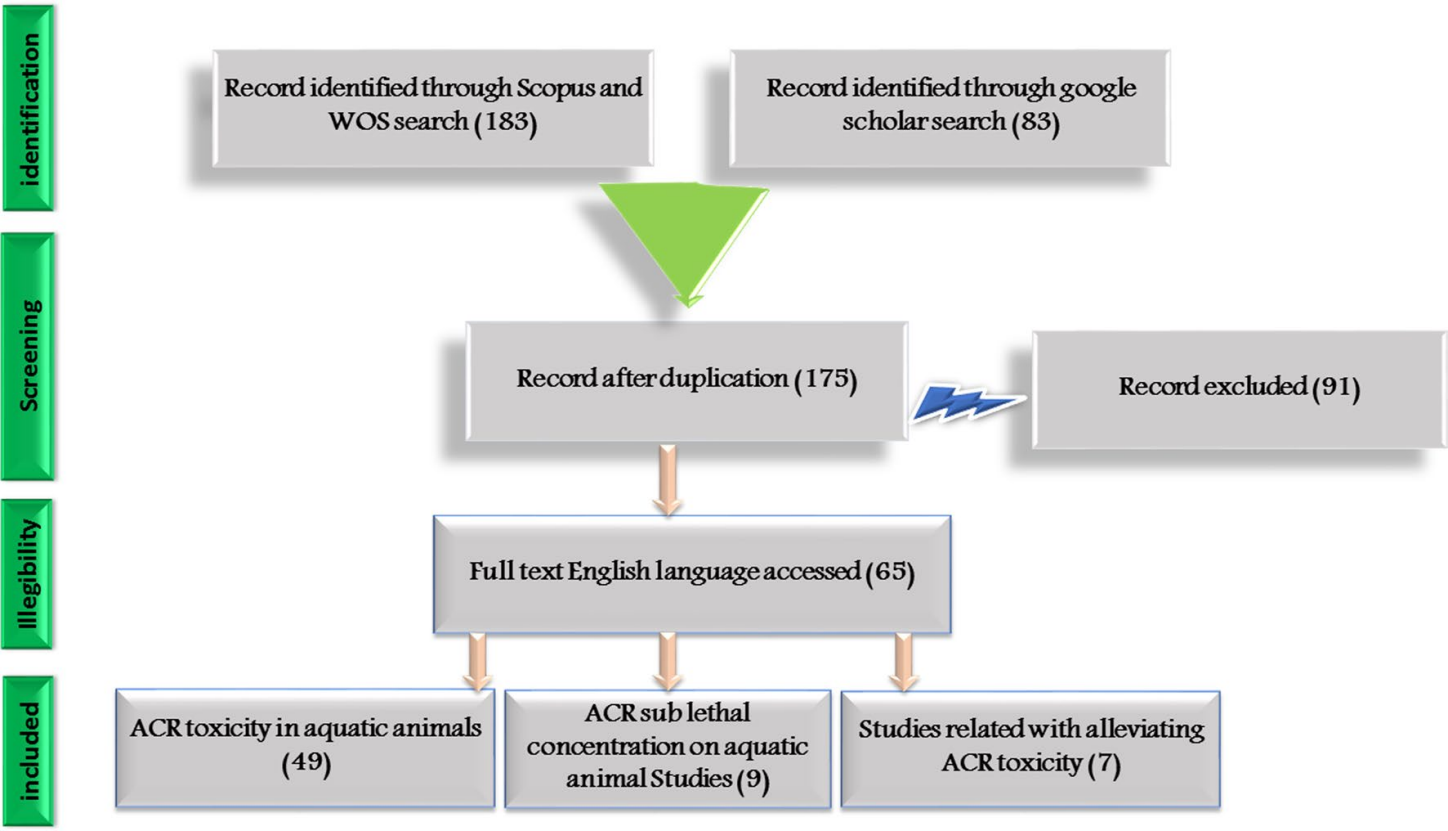
Prism flow flow-chart for the selected research criteria

These searches returned a total of 183 studies from Scopus and WOS as well as other 83 studies from Google Scholar. All of the obtained results were then inspected using the method of Preferred Reporting Items for Systematic Reviews (PRISMA; Fig. 1) (Rethlefsen et al. 2021) to additionally refine the search results by reading the title, abstract, and/or material and methods. The final screening left 65 publications that met the following criteria: (1) the hazard effects of ACR on fish and shellfish production and health (49 studies); (2) the ACR sub-lethal concentration on fish rearing water (9 studies); (3) the protective potential effects of some feed additives against ACR toxicity in fish (7 research items). The main obtained results are summarized in Tables 2 and 3. The current study focuses on several safe feed additives (acids, pigments, and salts) and other phytochemical as strategies to reducing ACR toxicity in fish.

### Acrylamide structure and resources

**Fig. 2:**
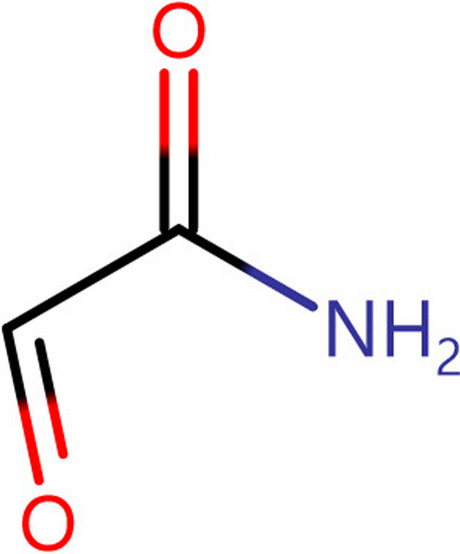
Chemical structure of ACR

ACR (CH_2_ = CHC(O)NH_2_) is a type of organic molecule (Fig. 2). It is a white, odorless solid that is soluble in water and a variety of organic solvents (alcohol and acetone) and insoluble in benzene and heptane (Stadler and Scholz 2004). In chemistry, acrylamide is a primary amide that has been substituted with vinyl group (CONH_2_) (Sharma et al. 2013). Acrylamide is found to be stable at ambient temperature but may polymerize aggressively near its melting point (84.5 °C) or when exposed to ultraviolet (UV) radiation (Faroon et al. 2009).

Acrylamide is mostly present in plant-based meals that are dense in carbohydrates such as grain products, or coffee (Yu et al. 2023). ACR does not synthesize, or is formed only in low quantities in dairy, meat, or fish products (Fonger et al. 2014). ACR is more likely to accumulate when higher temperatures are used in the cooking process for longer periods of time (Lentz et al. 2008). Besides, trace levels of acrylamide contaminants could be detected in some treated drinking water from natural water streams when polyacrylamide is used to purify water (Fan et al. 2023). The permissible detectable levels of ACR found in water resources and fish flesh are displayed in Table 1. Most acrylamide exposures are derived from consumed food, with a far smaller proportion from drinking water (Jia et al. 2017). The majority of acrylamide leads to environmental pollution as a contaminant from polyacrylamide products when polyacrylamide is employed in treatments of the waste water or industrial effluents (Mehana et al. 2020; Shanker and Seth 1986).

**Table 1:** Permissible limits of ACR in water and fish products

Product/source	Unit	ACR level		Reference
		Minimum	Maximum	
Water treatments	mg L^−1^	0.5	600	Hays and Aylward (2008)
Drinking water	mg L^−1^	0.0	1	Fallahzadeh et al. (2017)
	μg L^−1^	0.1	0.5	WHO (2011)
	ng L^−1^	100	500	Backe et al. (2014)
River and tap water	μg L^−1^	0	5	WHO (2003)
Fish and seafood products	μg kg^−1^	30	39	FAO and WHO (2002)
Takeaway fish-based meals		0	10	Sirot et al. (2019)
Fish-based ready meals		0	14	Pacetti et al. (2015)

Furthermore, ACR rapidly migrates to ambient water because of its broad range of applications and high hydrophilicity (Aras et al. 2017; Tepe and Çebi 2019). Although polyacrylamide is non-toxic, its monomer ACR has been found to be neurotoxic and carcinogenic (Exon 2006; Spencer et al. 2018). Thus, contaminated water with ACR is a threat to aquatic wildlife.

### Acrylamide formation and contaminated ecosystem

ACR is a synthetic organic compound that is extremely carcinogenic, toxic, neurotoxic, and reprotoxic (Hays and Aylward 2008). ACR contamination in water occurs as a result of its release from various industrial operations such as waste water treatment, pulp and paper preparation, agriculture, oil drilling, dam construction, cement, herbicide, cosmetics, soap, chalk, skin care products, adhesives, dyes, explosives, printing inks, latex, and mining and mineral production (Tepe and Çebi 2019). Other suppliers, such as acrylamide-based grouting and waste paper recycling, may also result in the discharge of acrylamide into rivers, ultimately resulting in water contamination (Kusnin et al. 2015). Moreover, acrylamide is usually found in drinking water as a consequence of leaching during the purification process (van Dijk-Looijaard and Van Genderen 2000). Specifically, the primary source of ACR pollution in drinking water is the improper application of polyacrylamide flocculants dragging residual amounts of ACR monomer (Tepe and Çebi 2019). In overall, the maximum permitted dosage of polymer is 1 mg/L (Tepe 2015).

The International Agency for Research on Cancer (IARC) categorized ACR as a carcinogenic molecule followed by the “2A Group” (McDonald 1995). Because of their proven aquatic toxicity, oil-based ACR products are unsuitable for application in aquatic environments (WHO 2003). The lethal concentrations of ACR in various fish species are illustrated in Table 2. Acrylamide caused cancer in experimental animals when animals were exposed to extremely high doses of acrylamide (Rice 2005). Specifically in rodents, acrylamide is transformed to glycidamide, which induces DNA alterations and damage (Besaratinia and Pfeifer 2007). Besides, ACR does not form, or forms at lower levels, in dairy, meat, and fish products (Tareke et al. 2002). It has been shown that high ACR pollution in the environment leads to higher accumulation in fish flesh depending on exposure time. According to Petersen et al.’s (1985) findings, exposed fingerling rainbow trout at 0.388 and 0.710 mg L^−1^ ACR levels resulted in about 72% of the ACR level accumulating into tissue and the remaining 28% consumption dosage removal out of the fish body *via* gills, urine, and bile exert after 2 h.

**Table 2:** ACR lethal concentration (LC50) in several fish species

Species	LC50 level	ACR forms	Reference
*Mytilus galloprovincialis*	0.3–10 mg L^−1^	Acrylamide combined polyacrylamide	Larguinho et al. (2014a)
*Danio rerio* embryos	1000 mg L^−1^		Moura et al. (2019)
Rainbow trout	110 mg L^−1^	Acrylamide monomers	Krautter et al. (1986)
Fathead minnows	120 mg L^−1^		
Bluegill fish	100 mg L^−1^		
Mysid shrimps	78 mg L^−1^		
*Rasbora heteromorpha*	460 mg L^−1^ for 24 h; 250 mg L^−1^ for 48 h; 130 mg L^−1^ for 96 h	Acrylamide	EPA (1994)
*Poecilia reticulate*	35 mg L^−1^		
*Mytilus galloprovincialis*	411 mg L^−1^		Larguinho et al. (2014a)
*Heteropneustes fossilis*	86.81–104.13 mg L^−1^		Shanker and Seth (1986)
*Danio rerio* embryos	100–585 mg L^−1^		Spencer et al. (2018)
*Oreochromis niloticus*	8.96 μg L^−1^		Gopika et al. (2018b)
*Mysidopsis bahia*	109 mg L^−1^ for 48 h		Bolan et al. (2020)

The overall degradation of acrylamide has been shown to vary between 480 and over 1100 h depending on the source of water samples such as tap, river, and lake (Babuji et al. 2023). The combination of sunshine and glyphosate has been found to reduce ACR breakdown (Ver Vers 1999). Also, mechanical degradation of small amounts of ACR in water could be induced by shaking, pumping, injection, and passage water through porous media (Aktağ et al. 2022). The fundamental issue with the mechanical degradation process may be the irreversible alteration of the polymeric component (Valipour and Montazar 2012). Furthermore, the high level of ACR may be released into the aquatic environment after being used in agricultural areas, causing a risk to human health and threatening aquatic wildlife (Mekawi et al. 2023). Thus, estuarine or marine water may keep ACR stable for a substantially longer period of time compared with freshwater (Friedman et al. 1995). Moreover, the Croll et al. (1974) study on acrylamide degradation in Hackensack River, New Jersey, reported that a 10-mg L^−1^ ACR level required 12 days to completely degrade. While in the Thames River in England, it was illustrated that 8 g L^−1^ ACR pollution needs 10 days to decompose entirely (Croll et al. 1974). In addition, Shanker et al. (1990) demonstrated that ACR levels exceeding 2 mg L^−1^ are not easily degraded in water; thus, these levels are considered hazardous for aquatic organisms. Furthermore, it was found that the optimal water quality conditions for acrylamide biodegradation have been described as pH 6–8 and water temperature 15–30 °C (Zamora et al. 2015).

Moreover, ACR lethal concentration tests have been determined on several fish and crustacean species. The LC50 levels were estimated and identified as 160 mg L^−1^ for common water fleas, 110 mg L^−1^ for *Oncorhynchus mykiss*, 120 mg L^−1^ for *Pimephales promelas*, and 100 mg L^−1^ for *Lepomis macrochirus*; furthermore, opossum shrimps presented the high sensitivity to LC50 value of 78 mg L^−1^ (Krautter et al. 1986). The ACR LC50 values for *Rasbora heteromorpha* (harlequin fish) were 460 mg L^−1^ for 24 h, 250 mg L^−1^ for 48 h, and 130 mg L^−1^ for 96 h, and found to be 35 mg L^−1^ for 7 days for *Poecilia reticulate* (guppy) (Torres et al. 2014). In contrast, marine crustaceans are showed highly tolerant to ACR contamination and had larger LC50 values (411 mg L^−1^) (Larguinho et al. 2014b). Nevertheless, the highest residual ACR levels estimated in water are 5–460 times lower than ACR amounts accumulated in commonly consumed tissues (Tepe 2015).

### Hazards of ACR in fish and crustacean

ACR is classified under two categories: polymeric, which is non-toxic, and monomeric, which is extremely harmful to the welfare of mammals and fish with carcinogenic, teratogenic, and neurotoxic effects (Park et al. 2021). It is well known that adult gills and embryonic yolk skin of fishes are the main organs that absorb calcium ions from the aquatic system (Lin and Hwang 2016). ACR may bind to redox-sensitive proteins (cystine) and disrupt calcium signaling genes, resulting in disruption of calcium homeostasis (Flik et al. 2009). It is hypothesized that calcium signaling disturbance might possibly trigger cancer formation through inducing hormonal alters (Pratt et al. 2020). However, the absence of actual evidence requires additional investigation toward this idea. Moreover, the neurotoxic effects of ACR have been linked to changes in the gut-brain axis signaling pathway caused by increments in oxidative stress, as well as modulates in the NrF2 signaling pathway and inflammatory mediator levels (alteration in NF-kB signaling pathway) in both the gut and the brain, which are expected to result in neurobehavioral disorders such as abnormal swimming phenotypes, including freezing, looping, and erratic movement in adult zebrafish (Singh 2020). Thus, Singh (2020) investigated that exposed adult zebrafish to several concentrations of ACR (1 mM and 2 mM) markedly induced sudden mortalities at 24 h and 48 h of exposure, while rearing zebrafish in ACR-contaminated water for 3 days at level 0.75 mM induced significant abnormalities in motor activities and oxidative stress as compared to the untreated group. Furthermore, Park et al. (2021) exhibited that ACR could induce developmental toxicity, which manifested as yolk retention, scoliosis, swim bladder deficit, and body curvature as well as reduced locomotor activity, as estimated by swimming speed and distance traversed. In addition, intoxicated zebrafish rearing water with 100 mg L^−1^ ACR significantly reduced the width of both the brain and spinal cord, suggesting neuronal damage (Park et al. 2021).

At the same context, exposed adult zebrafish to ACR (0.65–3.0 mM) for 72 h significantly induced severe abnormal behavior signs, which is associated with a “depression-like” phenotype (Faria et al. 2018). Besides, the downregulation of regeneration-associated genes and the upregulation of oligodendrocyte and reactive astrocyte markers, as well as changes in the expression of genes involved in presynaptic vesicle cycling, were found to be in combination with ACR exposure (Faria et al. 2018). Moreover, ACR-treated groups exhibited substantial alterations in the brain proteome and generated adducts with specific cysteine residues of particular proteins, some of which are required for presynaptic function (Faria et al. 2018).

Besides, ACR-exposed catfish for 96 h to LC50 level (133 mg L^−1^) considerably caused numerous aberrant behavioral indications and clinical and postmortem reactions. Furthermore, ACR-exposed catfish at the same level decreased red blood cell counts, hemoglobin level, and packed cell volume, resulting in anemic responses (Ibrahim and Ibrahem 2020). The anemic reaction may be induced by ACR’s degradation or suppression of erythrocyte formation (Ramesh et al. 2014). In the same study, malondialdehyde levels were considerably higher, in contrast with the glutathione, superoxide dismutase, and total antioxidant capacity were markedly smaller. Notably, the DNA fragmentation assay revealed a distinct laddering pattern in brain tissues affected by ACR (Ibrahim and Ibrahem 2020). Adduct formation with decreased glutathione and increased hydrogen peroxide generation results in higher levels of lipid peroxidative products and carbonyl content, and lower enzymatic and nonenzymatic antioxidants with a reduction in acetylcholinesterase (AChE) activity in the brain tissues (Petersen et al. 1985).

In comparison to the untreated group, rearing the land snail, *Theba pisana*, in polluted water with 1/20 LC50 ACR (ãpproximately 2.28 μg g^−1^) for 2 weeks substantially increased lipid peroxidation levels and the activity of catalase and glutathione-S-transferase, cell death, and hemocyanin content, while significantly decreasing DNA and reduced glutathione concentrations, phagocytic activity, lysosomal membrane stability, lectins, O_2_ generation, peroxidase, and phenol-oxidase levels (Radwan et al. 2019). Whereas, after 1 week of recovery, the majority of the observed markers in exposed snails were permanent and not reversible to normal values (Radwan et al. 2019), while Petersen et al. (1987) showed that exposed trout fish to 50 mg L^−1^ ACR for 15 days impaired swimming performance and caused rapid death. These irregular behavioral changes were discovered to be associated with ACR dose-related lesions in rainbow trout gills and the liver.

Owing to histopathological examination studies, Jia et al. (2017) investigated that acute ACR (2 mM for 36 h) exposure of zebrafish resulted in a significant decrease in motility and a loss of color-preferential swimming behavior (zebrafish preferred blue illumination to white, and white illumination to red). Hence, histopathological analysis of acrylamide-treated zebrafish eyes confirmed the results that the acrylamide exposure induced retinal damage. In addition, Haasch et al. (1992) reported that the hepatic CYP1A1 mRNA transcription increments were significantly altered by 50 ppm acrylamide monomer, whereas CYPI Al isozyme levels and EROD activity were downregulated. Sen et al. (2012) suggested that acrylamide treatment alone could have the possible isozyme selective inactivation or decreased translation of the CYP1A1 mRNA leading to reduction in isozyme levels consequently led to high transcription of the CYP1A1 gene in rainbow trout. Recently, Yue et al. (2021) reported that exposing embryos of *Oryzias melastigma* to various concentrations of ACR (from 0.1 to 10 mg/L) for 21 days substantially decreased hatching rate and prolonged hatching time, resulting in developmental delay, teratogenesis, and motility deficits in larvae. Transcriptome studies supported that these hazard effects might be linked to ACR-induced hypoxia and neurotoxicity. Moreover, the hazards of ACR in certain fish species are offered in Table 3.

**Table 3:** The hazards and toxicity of ACR to aquatic animal species

Species	Dose	Exposure	Toxic effects	References
*Oreochromis niloticus*	8.96 μg/L	24, 48, 72, and 96 h	↑ SOD, CAT, and GSR levels ↓ LP and hydrogen peroxide concentrations ↓ acetylcholinesterase within the muscular tissue Induced histological damages (such as muscular atrophy, vacuolization, thick bundle formation, and leukocyte infiltration) in muscular tissue	Gopika et al. (2018a)
Adult zebrafish	12.8 mg/kg wet weight	30 days	Induced splenic damages (such as cyst formation, hemorrhage, and inflammation) Depressed immune activation (such as depressed melano-macrophage center, activation of macrophages, and upregulation of major inflammatory cytokines)	Komoike et al. (2020)
Zebrafish larvae	2 mM	24 h	↑ toxicity in BRF41 cells ↑ the upregulation of glutathione S-transferase pi 1 gene (gstp1) ↓ GST activity	Komoike and Matsuoka (2019)
*Carassius auratus*	50 to 200 mg/L	96 h	↑ the total DNA strand breakage ↑ the erythrocytic nuclear abnormalities ↑ the hepatic cytochrome P4501A (CYP1A) upregulation ↑ GST activity ↑ histopathological signs to pancreatic acini compared to the lessons found within the hepatic parenchyma ↑ hepatic tissue alterations under acute exposure	Larguinho et al. (2014b)
Rainbow trout	25 to 50 mg/L	14 days	↑ many hepatocytes histological lesions (such as necrosis around the central vein) ↓ ethoxyresorufin-O-deethylase activity within hepatic microsomes	Petersen and Lech (1987)
Zebrafish larvae	1 mM	3 days	↓ cognitive behavior ↓ oxidative responses ↑ microglia level and induced neuronal apoptosis ↓ cathepsin-B (CAT-B) translocation	Sharma and Kang (2020)
Adult zebrafish	0.75 mM	72 h	Induced mild-to-moderate gait behavior abnormalities ↓ the GSH levels in the brain	Faria et al. (2019b)
Zebrafish embryos	0.5, 1.0, 1.5, 2.0, 2.5, 3.0, 3.5, 4.0, and 5.0 mM	24 h	↓ the upregulation of *nkx2.5*,* myl7*, and *vmhc* ↓ the capacity of cardiomyocyte proliferation ↓ myocardial cells and endocardial cells disordered of atrioventricular canal (AVC)	Huang et al. (2018)
Zebrafish embryos	0.5 and 1.0 mM	7 days	↑ thickened the vein chamber wall ↓ the trabeculae within the ventricular chamber ↓ the ventricular shortening fraction and spatial dimension ↑ the Notch signal in myocardium during cardiac maturation ↓ The re-distribution of N‑cadherin and failed to coordinate cardiomyocyte interactions between the myocardium layers due to the lack of delaminated cardiomyocytes Induced subcellular pathological states (for instance, disarrayed myofibrils and abnormal morphology of mitochondria) Upregulated the transcription of some cardiac-specific factors (*hand2* and *nkx2.5*) in hearts	Huang et al. (2019)

## New strategies toward the elimination of ACR hazards

### ACR biodegrading process using Bacteria

Natural bacterial populations have been discovered in ecosystems to be capable of degrading existing contaminants and increasing in number under pollution environment (Mehana et al. 2020). When a contaminant is degraded, the biodegradative population decreases as a result of the contaminated element (Sharif et al. 2023). The treatment byproducts are generally nontoxic compounds such as water, carbon dioxide, and cell biomass (Abatenh et al. 2017; Farag et al. 2021). The aerobic bacteria showed a higher ability to degrade ACR in freshwater with a half-life of 55–70 h, after acclimatization for 33–50 h (Charoenpanich 2013). ACR has been revealed to be stable slightly longer in estuarine or salt than in freshwater (Peng et al. 2016). The produced biomass including aerobic bacteria, phytoplankton and zooplankton, attends as a natural feed resource for the fish (Naiel et al. 2021b). ACR is offered to totally biodegrade *via* microbes existing in water for 8 up to 12 days. *Pseudomonas* sp., which is naturally present in the ecosystem, had a higher biodegradation capacity toward ACR (4 g L^−1^), producing crylic acid and ammonia (Nyyssölä and Ahlgren 2019). Amidase was also exhibited to be the appropriate enzyme for the degradation of ACR and other short chain phosphoramidites such as formamide and acetamide but not on ACR derivatives, meth-acrylamide, and N, N-methylene bisacrylamide (Bao et al. 2009). Besides, *Pseudomonas* sp. was found to be able to produce energy and obtain carbon from the degradation of ACR (Patial et al. 2022). In addition, *Pseudomonas stutzeri* might be applied in waste water treatment since it has a high capacity to remove ACR at lower dosages than 440 mg L^−1^ under aerobic conditions (Wang and Lee 2001). It was discovered that acclimating bacteria or its byproducts (specifically, lactic acid bacteria, yeast, and cell-free extracts) to aquatic environments improves their ability to degrade ACR (Albedwawi et al. 2021). ACR hydrolysis at 10–20 ppm in river water took approximately 12 days with non-acclimated bacteria but just 2 days with acclimated bacteria (Shen et al. 2012).

Furthermore, several bacterial species isolated from natural streams such as *Enterobacter aerogenes*, *Kluyvera georgiana*, *Klebsiella pneumoniae*, and *Enterococcus faecalis* shown higher removal capacity to degrade ACR up to 5000 ppm at trophic temperatures and could degrade various aliphatic amides, particularly short- to medium-chain length, but not amide byproducts (Buranasilp and Charoenpanich 2011). While in industrial waste water treatment, *E. aerogenes* had a higher removal capacity against ACR and ammonia than a mixture of organisms (Charoenpanich 2013).

Recently, *Ralstonia eutropha TDM-3*, a denitrifying bacterium isolated from a water treatment system connected with the production of polyacrylonitrile fiber, displayed a higher capability to an ingested higher level of ACR up to 1446 mg L^−1^, beyond which it was not dangerous (Cha and Chambliss 2011).

In conclusion, the global interest in environmental issues is rising, as are the expectations for sustainable and controlled processes that do not substantially hazard the ecosystem. Biodegradation is a traditional technique for removing undesirable organic chemicals to undetectable quantities or below regulatory-agreed-upon limits. Consequently, it is critical to identify more useful bacterial strains capable of removing ACR from the water environment while causing no damage to aquatic welfare sustainability.

## Nutritional antioxidant

### Fatty acids

Oxidative stress has been associated with numerous chemical-induced cellular injuries (El-hameed et al. 2021). Oxidative stress occurs when there is an imbalance between the production and scavenging of free radicals, which is associated with neurodegenerative disorders (Naiel et al. 2020b). Absorption of ACR from the gastrointestinal tract or *via* the gills with reduced glutathione might produce higher levels of lipid peroxidative (LPO) and carbonyl groups, and decreased enzymic and non-enzymic antioxidant activities with reduced AChE production in the brain (Khafaga et al. 2020). Acetylcholine is an essential neurotransmitter whose activity is reliant on the creation of AChE enzyme that metabolizes it (Ismael et al. 2021). Inhibiting AChE may disrupt metabolic and neurological function, as well as cause varied membrane permeability and ionic refluxes (Naiel et al. 2020d). ACR-induced membrane sensitivity to LPO may result in a decrease in adenosine triphosphatase (ATPase) activity. Any disruption in ATPase activity affects membrane stability by inducing changes in neuronal homeostasis and electrophysiological energetics (Farag et al. 2021; Lakshmi et al. 2012). Thus, ACR accumulation causes neuronal cell death by altering the ratio of free radicals to antioxidants (Jia et al. 2017).

Fish oil, found mostly in herring and salmon, includes *n-3* essential fatty acids (EFA), particularly eico-sapentaenoic acids (EPA) and docosahexaenoic acids (DHA), which are nutritional antioxidants and have been shown to be preventive elements against neurological diseases (Kinsella 1990). Fish oil has been proven to have anti-cancer, anti-inflammatory, and heart-protective properties (Al-Gabri et al. 2021; Weitz et al. 2010). Moreover, previous research has revealed that fish oil has neuroprotective effects due to its antioxidant properties (Panahi et al. 2019). For instance, Lakshmi et al. (2012) exhibited that enriched rat diets with fish oil have a neuroprotective impact on ACR-induced neurotoxicity by decreasing oxidative stress and apoptosis and upregulating the *HSP27* expression. The higher omega-3 fatty acid content of fish oil promotes vitamin E incorporation into cellular membranes and prevents lipid peroxidation caused by increased membrane PUFA levels (Mabile et al. 2001). In addition, according to Saif-Elnasr et al.’s (2019) findings, administration of fish oil and/or SeNPs may be helpful in avoiding nephrotoxicity induced by cisplatin and radiation during the treatment of different tumors. As a result, it seems that fish oil improved resistance to free radical damage caused by ACR administration and increased overall antioxidant capacity (Lakshmi et al. 2012). Despite the fact that there is no available scientific data to support the preventive role of fish oil against ACR hazards in aquatic fish, further research is required to validate this important topic.

### Pigments

The entire cellular antioxidant defensive mechanism is critical in the removal of numerous toxic harmful effects (Seidavi et al. 2021). Medicinal herbs are well known for their antioxidant properties and are still extensively applied as an effective therapeutic medicine resource (Farouk et al. 2021). The potential pharmacological aspects of these natural products derive from their higher contents of several bioactive compounds such as polyphenols, carotenoids, lignans, alkaloids, glycosides, cyanogenic, and terpenes (Durazzo et al. 2018; Gharib et al. 2022; Naiel et al. 2021a). Specifically, carotenoids are the main group of over 750 naturally synthesized pigments that are produced by algae, plants, and photosynthetic microorganisms (Tapiero et al. 2004). Lycopene (Ly) is an acyclic non-provitamin retinol carotenoid, as well as it is found in red-pigmented vegetables and fruits such as watermelon, tomatoes, pink guava, pink grapefruit, apricots, and pomegranate (Holick et al. 2007). It must be mentioned that Ly cannot be generated inside the body and that its bioavailability may be reduced by aging and certain pathological changes; thus, it must be supplied on a daily feed (Petyaev 2016). It is mainly found in trace quantities in the hepatocytes, adrenal gland, and brain tissues (Moran et al. 2013). Ly is strongly supposed that it might have neuroprotective effects in the central nervous system (CNS), because of its capability to permeate the blood–brain barrier (Rao and Rao 2007).

Several recent studies have indicated that lycopene-enriched fish diets, which have high antioxidant properties, effectively reduce oxidative damage induced by various toxicants or other kinds of abiotic conditions (Dawood et al. 2020). For instance, owing to the findings of Abd El-Gawad et al. (2019), supplementing the diet with Ly at a level of 400 mg/kg for 60 days might boost immunological response and sustain antioxidant defensive mechanisms in yellow perch. According to Farouk et al. (2021), oral treatment of Ly offers sufficient protection against the neurotoxicity of ACR on rat brain tissue structure and functions through regulation of oxidative and antioxidant activities. Besides, Fatma and Rabab (2019) proved that Ly attenuates the ACR-induced hepatocyte damage due to its high antioxidant properties. Owing to fish investigations, Ly helps fish stay healthy by boosting their immunological and antioxidative responses (Dawood et al. 2020). Because of its protective function *via* scavenging the excessive reactive oxygen species (ROS) mechanisms, fish diet inclusion with lycopene was shown to be mitigating oxidative stress (Wertz et al. 2004). It may also promote the expression of antioxidative-related genes and metabolic pathways involving phase II detoxifying enzymes (Lian and Wang 2008). Ly is a well-known, extremely effective scavenger of single t-oxygen (^1^O_2_) and other stimulated reactive oxygen molecules. During the ^1^O_2_ quenching process, the energy of generated ^1^O_2_ is transported into the lycopene molecule, converting lycopene to the energy-rich triplet form. Conversely, other generated free radicals such as OH, NO_2_, or peroxy-nitrite could induce oxidative degradation of the lycopene molecule. So, Ly might protect lipids, proteins, and DNA against the *in vivo* oxidation (Yonar 2012).

### Salts

Acrylamide is a carcinogenic and neurotoxic compound produced in heat-processed starchy foods. ACR is produced during the heating process of dietary components as a byproduct of the Maillard reaction induced between decreasing sugars and amino acids (Chen et al. 2016). Generally, heat-processed commercial protein-rich foods, for instance fish, meat, and chicken, often contain less ACR levels than carbohydrate-rich meals, such as French fries, potato or tortilla chips, grains, and baked products (Açar et al. 2012). The initial reaction step is thought to involve the creation of a primary base. The reaction begins with the binding of nucleophilic asparagine to the di-carbonyl molecules partly positive carbonyl carbon, which is followed by the loss of a proton from nitrogen and the binding of a free proton to oxygen (Mottram et al. 2002). Furthermore, Becalski et al. (2003) examined whether ACR might be produced *via* the rearrangement of nitrogen-based compounds found in cooked meals. As a result, finding an efficient method to eliminate ACR production in heat-processed foods is an important concern in the food manufacturing industries.

It is important to note that anions as well as cations have a significant impact on ACR synthesis during food manufacturing. For instance, sodium chloride inhibits ACR production via a variety of methods. It has the ability to form complex compounds by binding amine groups with certain intermediates created from the Maillard reaction (Lindsay and Jang 2005). Recently, it was proven that positive charge ions alter the pathway of the Maillard reaction by boosting the removal of water from glucose (Ciesarova et al. 2006). Besides, Na^+^ ion has been discovered to binding with asparagine to prevent ACR production (Omotosho 2015). Finally, the addition of sodium chloride salt may diminish water activity, resulting in less oil absorption and therefore promoting acrolein production (Omini et al. 2019).

In recent years, many methods for decreasing acrylamide production in heated meals have been suggested, including divalent cations, such as calcium salts. Chen et al. (2016) suggested that enriched shrimp fried chips with 0.1% calcium lactate resulted in the highest reduction of ACR formation level. According to Kukurová (2015) results, calcium chloride was attained to be the most effective via removing almost 90% of the ACR content. Also, sodium acid, sodium pyro-phosphate, and potassium di-hydrogen phosphate were highly efficient in removing nearly 75% from the total acrylamide level, followed by calcium lactate, sodium chloride, and potassium chloride, which resulted in a reduction of ACR content to 40–45%, and finally sodium and potassium hydrogen carbonates were found to be effective in removing nearly 30% from ACR content. The quantity and type of calcium substitutes applied had a strong impact on the formation of ACR in fish processed chips. As previously reported by Gökmen et al. (2007a) and Gökmen et al. (2007b), the inclusion of organic acid in cookie fish recipes may increase the formation of ACR as a result of sucrose hydrolysis to decreasing sugars level. Thus, the decreasing level of sugar in shrimp chips enriched with calcium citrate was higher than that recorded in shrimp chips supplemented with other calcium salts (Chen et al. 2016). The calcium carbonate and chloride salt were found to be effective in reducing ACR production, while adding up to 0.2% calcium propionate salt for food preservation resulted in an increase in ACR production of 90%. Therefore, enhanced fish-heat processed products containing cationic or anionic salts should not be the fish processing sector’s first option for eliminating ACR generation, while avoiding high processing heat or overcooking is a more effective and safe method of preventing ACR formation.

### Phytochemicals

Phytochemicals may be defined as a bioactive molecule found in many medicinal plants that is able to regulate metabolic functions and boost health status (Naiel et al. 2020a). They have a favorable impact such as antioxidant properties, promoted enzyme activity, and upregulated or downregulated specifically related genes (Naiel et al. 2020c). The antioxidant properties of herbs also played a vital role in preventing the harmful impact of ACR on fish health via improving the antioxidant enzyme activity and reducing the free radical concentration (Hassan et al. 2020). Phytochemical molecules could prevent ACR formation in fish flesh under high temperatures in several ways. The main pathway depends on activating the reduced glutathione activity, inhibiting the reactive oxygen formation, and reducing oxidative stress (Zhu et al. 2012). Also, herbs or their extracts may have an ameliorating effect due to phytochemical compounds that are simply oxidized, as well as the rate of oxidation and the oxidized products that are discovered to be reacted with asparagine (Bartoszek 2002).

Several cellular studies in animals or fish proved the potential protective role of herbs or their extracts against ACR hazards for natural phytochemical compounds such as eugenol, iso-eugenol, turmeric, polyphenol, flavonoids, N-acetylcysteine, gallate, and carnosic acid (Albalawi et al. 2018; Faria et al. 2019a; Krishnan and Kang 2019; Ning et al. 2021; Singh et al. 2019; Wang et al. 2021). Table 4 displays a collection of references that demonstrate the possible ameliorative effect of certain phytochemical substances against ACR toxicity. Besides, the phenol ion may create a powerful nucleophile, or electron giver, to the final chemical carcinogen’s electrophile. Moreover, the following essential functional groups are thought to be present in phenolics that allow them to serve as efficient electrophilic trapping agents: (1) one phenolic group must be present to react with catechol and decreased the pKa level. (2) At least one unsaturated substituent must be present in the aromatic ring in order for it to bend with reactive free oxygen. On the other hand, many studies examined the impact of different herbs and their extracts on ACR toxicity but found inconsistent results. It might depend on the antioxidants’ capacity to react with ACR intermediates, related chemicals, or ACR form itself, resulting in either decreasing or stimulating effects (Exon 2006). Thus, it is critical to conduct new experimental research on the efficiency of herbs or their extracts in reducing the toxicological risks of ACR on fish’s general health status and providing deep insights into cellular regulating activities under toxicity situations.

**Table 4:** A list of references for certain compounds that have a protective function against ACR toxicity in fish

Compounds	Source	Fish species	Dose	ACR level	Biological activity	References
Polyphenolic	*Morus nigra* leaves	Zebrafish larvae	1 mL/L (ethanolic extract)	0.75 mM per 5L	↑ CAT activity ↓ MDA level ↑ GSR activity was detected in brain tissue	Singh et al. (2019)
Carnosic acid	Rosemary and common sage leaves	Zebrafish larvae	10 μM	1–2 mM	↑ antioxidant activity toward photoreceptor cells ↑ activation of the antioxidant NRF2/ARE pathway	Albalawi et al. (2018)
Vitexin	Passion flower, *Vitex agnus-castus*, *Phyllostachys nigra* leaves, *Pennisetum millet*, and Hawthorn	Zebrafish larvae	10 μM	1–3 mM	↓ histological and behavioral changes in zebrafish larvae ↓ CDK5 expression ↓ pro-inflammatory mediators ↓ the loss of neuroplasticity markers ↑ antioxidant markers in larvae	Krishnan and Kang (2019)
N-acetylcysteine	Allium species	Adult zebrafish	0.3 mM	0.75 mM	↓ NAC uptake to the brain ↓ BBB permeability ↑ deacylation of NAC during the intestinal absorption	Faria et al. (2019a)
(-)-Epigallocatechin gallate and curcumin	Turmeric	Zebrafish larvae		1, 5, 10 mmol/L	↓ DNA damage Protected embryos in the growth and developmental stages	Wang et al. (2021)
Polyphenolic	Onion peel powder	*Oreochromis niloticus*	20 mg/L	8.00, 9.80, 9.75, and 17.00 mg/L	Attenuate the cytotoxicity and immunotoxicity in hepatocyte cells ↑ antioxidant protective mechanism	Elhassaneen and Abd Elhady (2014)
Triphala	*Amalaki Phyllanthus emblica*, *Bibhitaki*, and *Haritaki*	Zebrafish	3 mL	10 mmol/L	↓ necroptosis ↑ scavenging free radicals in the CNS	Ning et al. (2021)

## Conclusion

ACR is a highly water-soluble unsaturated organic polymer with a broad range of applications, including water purification, agricultural operations, paper production, and oil drilling. ACR is a neurotoxin carcinogenic, mutagenic, and reprotoxic compound for both humans and fish. The ACR could be generated in high-temperature processed food. Moreover, the ecological fate of ACR following deterioration or release into water streams has gained great attention. Because of its high solubility, ACR remains in water after treatment and is not easily absorbed by the soil. Besides, ACR is found to be biodegradation by bacteria in surface water. A contaminated ecosystem with ACR presented higher potential hazards in the behavior, reproduction, and nervous system of fish. In addition, ACR-contaminated water has deleterious effects on the early development of some fish species, for instance decreased hatching percentage, prolonged embryo hatching time, delayed larval development, and induced malformations. Transcriptome reports indicated that these harmful effects might be correlated with ACR-induced hypoxia and neurotoxicity. Though, treated fish exposed to ACR with a variety of fatty acids, salts, or phytochemical substances showed a high elimination rate against its toxicity. Meanwhile, biodegradation of ACR-contaminated environments by bacterial strains found in natural ecosystems provides an efficient solution to its ecological fate. When using fatty acids, salts, and phytochemical compounds to reduce ACR risks, both dosage and exposure duration of ACR levels should be taken into consideration. There is a need to identify the ACR threats to aquatic fish via further research efforts and to propose more applicable methods that may eliminate the risks in both fish and humans. Furthermore, this literature provides essential information on the toxicity of ACR in fish and crustaceans, which might be very useful in maintaining ACR’s aquatic environmental risk assessment.

## Data Availability

All related data were available under reasonable request from the corresponding author.

## Abbreviations

ACR: Acrylamide
UV: Ultraviolet radiation
IARC: The International Agency for Research on Cancer
LC50: Lethal concentration 50
NrF2: Nuclear factor erythroid 2–related factor 2
DNA: Deoxyribonucleic acid
AChE: Acetylcholinesterase
O_2_: Oxygen
CYP1 A1: Cholesterol side-chain cleavage enzyme
EROD: Ethoxyresorufin-O-deethylase
LPO: Lipid peroxidative
ATPase: Adenosine triphosphatase
EFA: Essential fatty acids
EPA: Eico-sapentaenoic acids
DHA: Docosahexaenoic acids
HSP27: Heat shock protein 27
PUFA: Polyunsaturated fatty acids
SeNPs: Selenium nanoparticles
Ly: Lycopene
CNS: The central nervous system
ROS: Reactive oxygen species
^1^O_2_: Single t-oxygen
OH: Hydroxyl group
NO_2_: Nitrogen dioxide
Na^+^: Sodium ion
pKa: Acid dissociation constants
NF-κB: The Nuclear factor kappa-light-chain-enhancer of activated B cells
mM: Millimolar
